# Depressive symptoms and their influencing factors among older adults in China: a cross-sectional study

**DOI:** 10.3389/fpubh.2024.1423391

**Published:** 2024-11-15

**Authors:** Xinyu Hu, Huan Liu, Qingwei Liu, Ting Yuan, Mengying Duan, Yang Luo, Jiahui Min, Guangliang Mei, Xiubin Tao, Bin Xuan, Ming Zhang

**Affiliations:** ^1^Department of Cardiovascular, The Second Affiliated Hospital of Wangnan Medical College, Wuhu, Anhui, China; ^2^Department of Hemodialysis, The First Affiliated Hospital of Wannan Medical College (Yijishan Hospital of Wannan Medical College), Wuhu, Anhui, China; ^3^School of Nursing, Shandong First Medical University, Jinan, Shandong, China; ^4^Department of Gynecology and Obstetrics Nursing, School of Nursing, Wannan Medical College, Wuhu, Anhui, China; ^5^Graduate School of Wannan Medical College, Wuhu, Anhui, China; ^6^School of Clinical Medical, Wannan Medical College, Wuhu, Anhui, China; ^7^School of Public Health, Wannan Medical College, Wuhu, Anhui, China; ^8^The Department of Party Affairs, The First Affiliated Hospital of Wannan Medical College (Yijishan Hospital of Wannan Medical College), Wuhu, Anhui, China; ^9^Department of Nursing, The First Affiliated Hospital of Wannan Medical College (Yijishan Hospital of Wannan Medical College), Wuhu, Anhui, China; ^10^School of Educational Science, Anhui Normal University, Wuhu, Anhui, China; ^11^School of Innovation and Entrepreneurship, Wanna Medical College, Wuhu, Anhui, China

**Keywords:** depressive symptoms, prevalence, risk factors, older adults, China

## Abstract

**Background:**

With the acceleration of population aging, depressive symptoms in older adults have become an urgent global public health issue. It is critical to understand how to prevent and intervene the epidemic of depressive symptoms. Several studies have reported the prevalence of depressive symptoms among older adults in urban and rural China, but there is a lack of cross-sectional studies on the prevalence of depressive symptoms among older adults in Anhui Province after the COVID-19 pandemic.

**Methods:**

Between December 2023 and February 2024, we used convenience sampling to randomly recruit 1,175 older people over 60 years old in Anhui Province, China. The PHQ-9 depressive symptom scale, frailty scale, and social frailty scale were used in the study. Logistic regression was used to analyze the association between frailty, social frailty, family health and depressive symptoms in older adults.

**Result:**

Of the 1,175 participants, 406 (34.6%) had depressive symptoms. Binary logistic regression results showed that Status of body pain (*p* < 0.001), Number of hospitalizations in the past year (*p* < 0.001), Status of social frailty (*p* < 0.001) and Status of frailty (*p* < 0.001) were highly correlated with depressive symptoms in older adults. While family health (OR = 0.53, 95% CI = 0.26–1.11, *p* = 0.092) was a protective factor for depressive symptoms in older adults.

**Conclusion:**

The prevalence of depressive symptoms among older adults is high in Anhui Province, China. Frailty, social frailty, and poor family health are associated with higher prevalence of depressive symptoms in older adults. Public health departments should pay more attention to the construction of mental health protection in the three environmental contexts of individual, family and society to promote healthy aging.

## Introduction

As the proportion of older adults in the population increases rapidly, countries around the world are facing the challenge of population aging. According to the seventh national census of China by the end of 2020, the number of older adults aged 60+ in China mainland had reached 264 million, accounting for 18.7% percent of the mainland’s total population (1). It is expected that by 2050, China’s older adult population will reach 400 million, accounting for one-third of the total population, meaning that China will enter the deep aging society by 2050 (2). According to data from the World Health Organization, due to the increase in life expectancy and the decline in the general fertility rate, the proportion of China’s population over 60 years old is expected to reach 28% by 2040 (3). Inevitably, China will soon face the tremendous pressure that diseases related to older adults put on the national healthcare system and families (4).

Older adults are a key group whose health problems need to be solved in the “Healthy China 2030,” which is related to the implementation of the national health strategy and the advancement of the healthy aging process (5). Depressive symptoms are a common mental health problem among older adults, which not only seriously reduces the quality of life and social participation, but also brings additional economic burden to medical and health institutions (6). In 2019, the Chinese government implemented the Healthy China Action Plan (2019–2030), an essential goal of which is to mitigate the rising trend of depression by 2030 (7).

Depressive symptoms are a major global public health problem that not only affects an individuals’ quality of life, but also imposes a high economic burden on society and families as a whole. Depressive symptoms are associated with physical health and cognitive function problems, shortage labor, and greater health care utilization (8). Depression is a common mental disorder affecting approximately 3.5 billion people worldwide (9). In China, the prevalence of depression among Chinese adults is approximately 6.9% (10), and about 40% of people aged more than 60 have suffered from depressive symptoms (11). According to research, approximately 30% of men and 40% of women aged 45 and above experience depressive symptoms (12). Depressive symptoms are considered the second leading cause of disability in China, and their prevalence is increasing rapidly with China’s GDP growth and population aging (13). Depression is the most common psychiatric disorder among older adults, with 8 to 16% of community-dwelling older adults experiencing clinically significant depressive symptoms (14). A meta-analysis showed that the prevalence of depressive symptoms among Chinese older adults was as high as 20.0% (15). The harm of depression in older adults is multifaceted. It may not only increase the risk of stroke but also cause immune system disorders and inflammation (16), unhealthy behaviors (17), reduced medication compliance (18), and the occurrence of hypertension and diabetes (19). The high prevalence of depressive symptoms in older adults has dramatically increased medical expenditures in China (20). Therefore, exploring the influencing factors of depressive symptoms in older adults and reducing their prevalence is crucial for the physical and mental health of older adults and the development of social public health.

As China’s aging population intensifies, the prevalence of frailty among older adults is on the rise. Frailty is defined by as a clinical geriatric syndrome, characterized by diminished strength, endurance and decreased physiological function, which is associated with increased risks of adverse clinical outcomes including falls, disability, hospitalization, mortality, and their prevalence is aggravated by age (21). Mounting prospective cohort studies indicates that frailty is associated with depressive symptoms. For example, the results of a prospective cohort study by Makizako et al. showed that frailty was an independent predictor of depressive symptoms among community-dwelling older people (22). In view of there was a paucity of large-sample cross-sectional studies demonstrating the relationship between frailty and depressive symptoms in older adults. Therefore, we conducted a cross-sectional study to investigate the association between frailty and depressive symptoms in older adults.

In recent years, the concept of “social frailty” has received increasing attention from academic circles. As an important dimension of frailty, social frailty was classically defined as the risk of individuals facing a decline in social functioning, which was characterized by a lack of social resource support sources, weak social networks, and low levels of social participation (23). Social frailty not only affects the process of healthy aging in China, but also poses serious threats to individuals, families, society and healthcare services systems. Studies have found that the prevalence of social frailty among community-dwelling older adults is estimated to be about 20% (24), which was significantly higher than the prevalence of physical frailty of about 10% (25), indicating that social frailty is a more serious social problem. Social frailty could negatively impact physical functioning in older adults (26) and, at the same time, could also lead to depression and disability in older adults (27). A four-year follow-up study found that social frailty was more closely related to depressive symptoms in community-dwelling older adults than physical frailty and cognitive frailty among the components of frailty (28). However, there is insufficient research on the relationship between depressive symptoms and social frailty among older adults in China. Therefore, exploring the association between social frailty and depressive symptoms in older adults could help accelerate the process of active aging and reduce the prevalence of depressive symptoms among older adults in China.

Family health has recently become a prominent focus in the field of modern geriatric medicine. In recent years, “family health” as a relatively new concept, has received more and more attention from scholars (29). On January 2, 2024, the Chinese National Health Commission issued the “Notice on Comprehensively Carrying out the Construction of Healthy Families,” which proposed to improve family health literacy and create a healthy family environment (30). Family systems theory underscores that the family is an organized system in which family members influence each other through their commitment to each other, exchange of information, emotions, and interests (31). Family health is “family-level resources that develop based on each member’s health, communication, abilities, and their interaction with the family’s physical, social, emotional, economic, and medical resources” (32). Family health covers a wide range of health-related social support and family interactions and resources (33), which helps improve individual health literacy and increase the level of healthy behaviors, thereby reducing the risk of disease. A healthy family can promote a sense of belonging among family members (34) and cultivate the ability of family members to take care of each other and assume life responsibilities (35). Family health research can enhance the cohesion and sense of belonging among family members, cultivate the ability of family members to care for each other and fulfill family life responsibilities, and ultimately contribute to the overall development of society (36). We therefore hypothesized that depressive symptoms in older adults would be significantly reduced as the intensity of support from spouses, children, or other family members increases. Therefore, family health can serve as an essential target for health intervention. However, there have been few studies on family health in older adults, and there are no previous studies on the relationship between family health and depressive symptoms in older adults.

As China is about to enter a profoundly aging society, special attention should be paid to the depressive symptoms of older adults. Old age is a more emotionally vulnerable time in a person’ s life. Physical aging in old age inevitably brings about many major losses, which have a significant impact on an individual’ s physical condition, emotions, and social status. Previous findings suggest that older adults are at increased risk for depressive symptoms. Depressive symptoms can take a toll on older adults’ physical and mental health, and the damage can last long or even get worse. There is an urgent need to explore the prevalence of depressive symptoms and related influencing factors in older adults to provide a reference for formulating effective intervention measures. However, to our knowledge, the effects of frailty, social frailty, and family health on depressive symptoms in older adults have not been previously studied. We hypothesize that frailty and social frailty may trigger depressive symptoms, leading to adverse health outcomes. Therefore, the purposes of this study are (1) to investigate the prevalence of depressive symptoms among older adults and (2) to explore the influencing factors of depressive symptoms in older adults.

In this study, we first analyzed the prevalence and sociodemographic characteristics of depressive symptoms among older adults in China. In the second step, we investigated the risk factors and protective factors associated with depressive symptoms in older adults and advocated promoting family health activities as “protective factors.” This study meets the needs of an aging society for the prevention and management of depressive symptoms in older adults. It can further help the government, society, and families develop effective psychological intervention measures to reduce the impact of depressive symptoms on the physical and mental health of older adults. Our findings identify critical elements for preventing and treating depressive symptoms in older adults, which are essential for reducing depressive symptoms and improving mental health in older adults.

## Materials and methods

### Study design and setting

This was a large-scale cross-sectional study that strictly followed the Strengthening the Reporting of Observational Studies in Epidemiology (STROBE) reporting guideline.

### Participants and data collection

**Figure 1:**
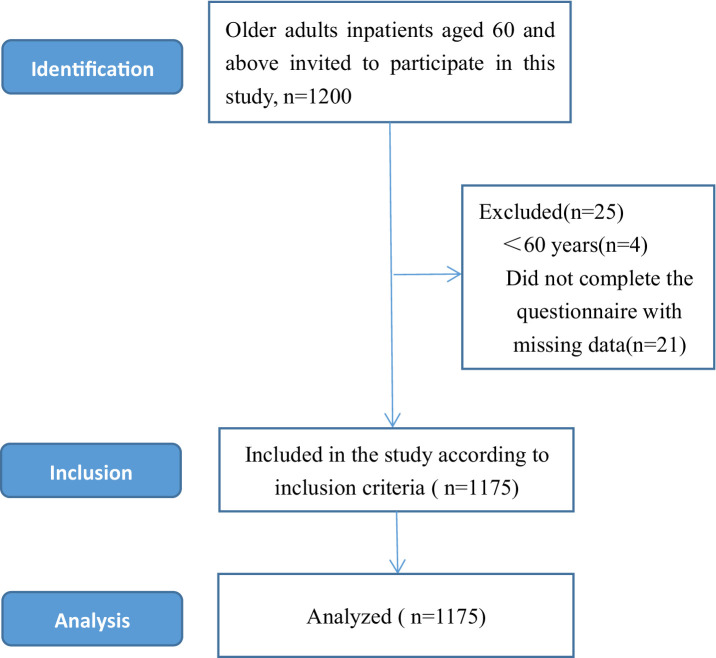
Sample selection process for this cross-sectional study.

Participants were recruited using convenience sampling in Anhui Province, China, between December 2023 and February 2024. Convenience sampling, also known as random sampling and chance sampling, is a non-probability sampling method in which the investigator randomly selects respondents at a specific time and in a particular location in a specific community to match the research topic. In this study, trained medical students from Wannan Medical College in Anhui Province administered a face-to-face questionnaire to each participant using a standardized questionnaire. The training for investigators included the purpose and significance of the investigation, the unified investigation terms, and related precautions that needed to be mastered. Based on this cross-sectional study design, participants aged 60 years and above, without deafness or dementia/neurological or mental illness, and who voluntarily participated in our survey met the criteria for filling out the questionnaire. Participants were completely voluntary, and the participants were informed in detail about the purpose, methods, procedures and confidentiality of the survey data before participating in the cross-sectional survey, and finally signed an electronic informed consent form. If participants no longer wish to cooperate in completing the survey, they may withdraw from the study at any time without any conditions. Participants carefully filled out the questionnaire, and data submission was conducted via a smartphone or tablet by a survey team member. Research team members strictly supervised the online data collection to achieve the desired research objectives. A total of 1,200 subjects were included in this study. After excluding invalid questionnaires, 1,175 valid subjects were finally included for data analysis, with an effective recovery rate of 97.9% (details are shown in Figure 1).

### Measurements

#### Demographic questionnaire

We developed the demographic questionnaire based on an extensive literature review and research purposes. The general sociodemographic information of this study includes age, gender, place of residence, education level, marital status, current chronic disease status, current work status, and self-evaluated physical pain status.

#### Assessment of depression symptoms

Depressive symptoms in older adults were assessed with the Patient Health Questionnaire (PHQ-9) (37). The PHQ-9 is a 9-item depression symptoms self-report instrument that evaluates the frequency of symptoms of depression symptoms in the past 2 weeks. Each of the 9 items was divided into a four-point degrees of the scale (0 = not at all; 1 = several days; 2 = more than half the time; 3 = nearly every day); the PHQ-9 total score range from 0 to 27, with higher scores indicated greater depression symptoms. All items of the PHQ-9 screening scale are consistent with the nine diagnostic criteria for depression in the Diagnostic and Statistical Manual of Mental Disorders (DSM-5) (37). In our study, a score of <10 was considered as no depressive symptoms, and a score of ≥10 was considered as depressive symptoms (38). In the current study, Cronbach’s alpha was 0.85.

#### Frailty

Frailty was indicated by fatigue sensation, resistance, free movement descent, coexistence of multiple diseases, weight loss (39). FRAIL scale consists of 5 items, each with “yes” and “no” options. The frailty final score was calculated cumulatively, with a total score of 0–5. Participants were considered “frail” if their total score was 3 or above. With a total score of less than 3 points was obtained, and participants were classified as “non-frail” (40). In the current study, Cronbach’s alpha was 0.83.

#### Social frailty

Social frailty was defined using the social frailty scale (41) (Help, Participation, Loneliness, Financial, Talk Scale, HALFT)comprising five components: (1) Have you been able to help a friend or family member in the past year? (2)Have you engaged in any social or recreational activities in the past year? (3) Have you felt lonely in the past week? (4) Is the income in the past year sufficient to survive for 1 year? (5) Are there people you chat with every day? Respondents answered each question with “yes” or “no,” the total score was 5 points, and respondents who answered three or more were considered to be in a state of social frailty. In the current study, Cronbach’s alpha was 0.82.

#### Family health

Participants’ family health was measured utilizing the Family Health Scale-Short Form (FHS-SF) (42). Each item was rated on a Likert scale with scores ranging from1 (strongly disagree) to 5 (strongly agree), with questions 6, 9 and 10 reverse-scored. Points were accumulated and the total score ranges from 1 to 50 points. The higher the score, the higher the levels of healthy family. In this study, participants who scored greater than or equal to 20 on the family health scale were classified as having a healthy family, while those who scored lower than 20 were classified as without healthy family. In the current study, Cronbach’s alpha was 0.82.

### Statistical analyses

Data analyses were performed using IBM Statistical Package for Social Science version 26.0 (SPSS Inc., Chicago, IL, United States). Demographic characteristics, depressive symptom scores, family health, frailty, and social frailty were presented as means, standard deviations (SD), numbers, and percentages. Chi-square tests were used to compare differences in categorical variables between depressive symptom groups and non-depressive symptom groups. In addition, logistic regression analysis was used to explore the relationship between depressive symptoms in older adults and general demographic characteristics, family health, and social weakness. The correlation among family health, social weakness and depressive symptoms was evaluated by Pearson’s correlation analysis. The Cronbach’s *α* coefficient of the scale in this study was 0.892.

## Results

### Demographic characteristic

A total of 1,175 participants were included in this study. The age of the participants ranged from 60 to 93 years old, with an average age of 71.73 ± 6.4 years old. These1175 participants were divided into four age groups: (1) 60–70 years old, 705 participants; (2) 71–80 years old, 367 participants; (3) 81–90 years old, 87 participants; and (4) >90 years old, 16 participants. 530 (45.1%) were male, and 645 (54.9%) were female. Place of residence had three categories: rural was 513 (43.7%), town was 432 (43.7%) and city was 230(19.6%). Detailed sociodemographic characteristics of participants are presented in Table 1.

**Table 1:** Sociodemographic characteristics of the participants (*N* = 1,175).

Item	Categories	N(%)	Depressive symptoms	No depressive symptoms	χ^2^/Z-values	*p*-values
Sex	Men	530(45.1%)	188(35.5%)	342(64.5%)	0.360	0.548
	Women	645(54.9%)	218(33.8%)	427(66.2%)		
Place of residence	Rural	513(43.7%)	169(32.9%)	344(67.1%)	1.056	0.590
	Town	432(36.8%)	154(35.6%)	278(64.4%)		
	City	230(19.6%)	83(36.1%)	147(63.9%)		
Age group	60-70 years old	705(60.0%)	205(29.1%)	500(70.9%)	30.026	<0.001
	71–80 years old	367(31.2%)	146(39.8%)	221(60.2%)		
	81–90 years old	87(7.4%)	46(52.9%)	41(47.1%)		
	>90 years old	16(1.4%)	9(56.2%)	7(43.8%)		
Suffering from chronic diseases	No	667(57.6%)	205(30.3%)	472(69.7%)	12.894	<0.001
	Yes	498(42.4%)	201(40.4%)	297(59.6%)		
Education level	Below elementary school	409(34.8%)	147(35.9%)	262(64.1)	7.640	0.177
	Elementary school	388(33%)	143(36.9%)	245(63.1%)		
	Junior high school	170(14.5%)	46(27.1%)	124(72.9%)		
	High school	105(8.9%)	40(38.1%)	65(61.9%)		
	Junior college	24(2.0%)	6(25.0%)	18(75.0%)		
	Bachelor and above	79(6.7%)	24(30.4%)	55(69.6%)		
Profession	Worker	155(13.2%)	64(41.3%)	91(58.7%)	15.014	0.091
	Farmer	541(46.0%)	183(33.8%)	358(66.2%)		
	Employees	80(6.8%)	36(45.0%)	44(55.0%)		
	Public employees	61(5.2%)	20(32.8%)	41(67.2%)		
	Medical staff	20(1.7%)	9(45.0%)	11(55.0%)		
	Self-employed	56(4.8%)	17(30.4%)	39(69.6%)		
	Freelancer	54(4.6%)	11(20.4%)	43(79.6%)		
	Teacher	46(3.9%)	13(28.3%)	33(71.7%)		
	Professional and technical	9(0.8%)	4(44.4%)	5(55.6%)		
	Other	153(13.0%)	49(32.0)	104(68.0%)		
Number of hospitalizations in the past year	0	470(40.0%)	87(18.5%)	383(81.5%)	134.505	<0.001
	1	321(27.3%)	104(32.4%)	217(67.6%)		
	2	202(17.2%)	106(52.5%)	96(47.5%)		
	≥3	182(15.5%)	109(59.9%)	73(40.1%)		
Marital Status	Single	50(4.3%)	19(38.0%)	31(62.0%)	44.599	<0.001
	Married	849(72.3%)	252(29.7%)	597(70.3%)		
	Divorced	36(3.1%)	25(69.4%)	11(30.6%)		
	Widowed	217(18.5%)	96(44.2%)	121(55.8%)		
	Other	23(2.0%)	14(60.9%)	9(39.1%)		
Status of physical pain	No physical pain	410(34.9%)	77(18.8%)	333(81.2%)	129.620	<0.001
	Mild physical pain	577(49.1%)	207(35.9%)	370(64.1%)		
	Moderate physical pain	147(12.5%)	88(59.9%)	59(40.1%)		
	Severe pain	41(3.5%)	34(82.9%)	7(17.1%)		
Type of employment	Manual workers	725(61.7%)	249(34.3%)	476(65.7%)	0.553	0.758
	Both manual and mental workers	333(28.3%)	113(33.9%)	220(66.1%)		
	Mental workers	117(10%)	44(37.6%)	73(62.4%)		
Frailty	No	947(80.6%)	240(25.3%)	707(74.7%)	183.061	<0.001
	Yes	288(19.4%)	166(72.8%)	62(27.2%)		
Social frailty	No	991(84.3%)	280(28.3%)	711(71.7%)	111.032	<0.001
	Yes	184(15.7%)	126(68.5%)	58(31.5%)		

### Factors associated with depressive symptoms in univariate analyses

In this study, the prevalence of depressive symptoms among older adults was 34.6% (406/1175). There were significant differences between age groups, chronic disease status, number of hospitalizations in the last 1 years, physical pain status, marital status, and medical insurance type (*p* < 0.01; Table 1).

### Correlations between frailty, social frailty, family health, and depressive symptoms

As shown in Figure 1, there was a significant positive correlation between frailty in older adults, social frailty and depressive symptoms (*p* < 0.01). In contrast, family health was significantly negatively correlated with depressive symptoms (*p* < 0.01).

### Binary analysis factors associated with depressive symptoms

**Figure 2:**
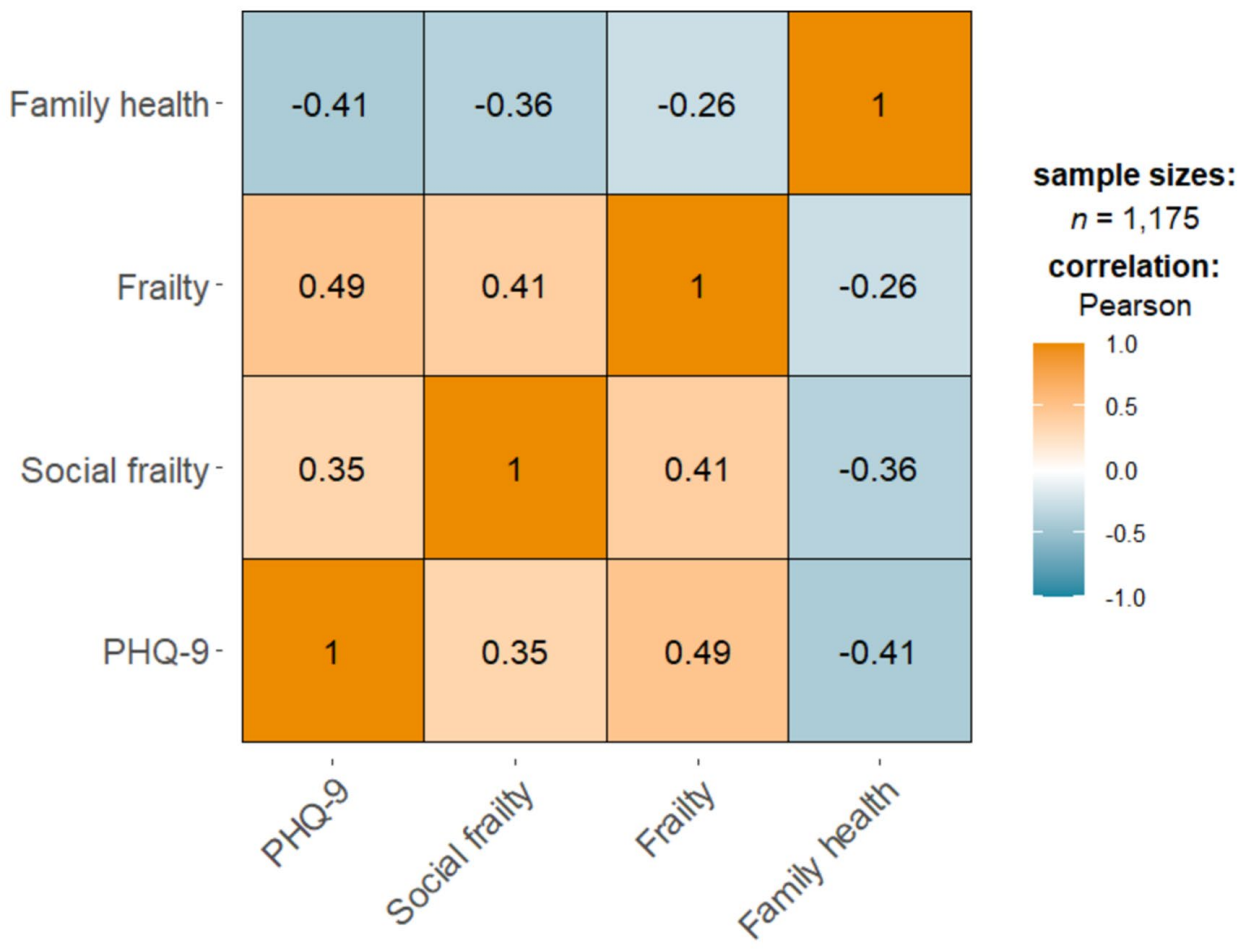
Correlations between frailty, social frailty, family health, and depressive symptoms.

**Figure 3:**
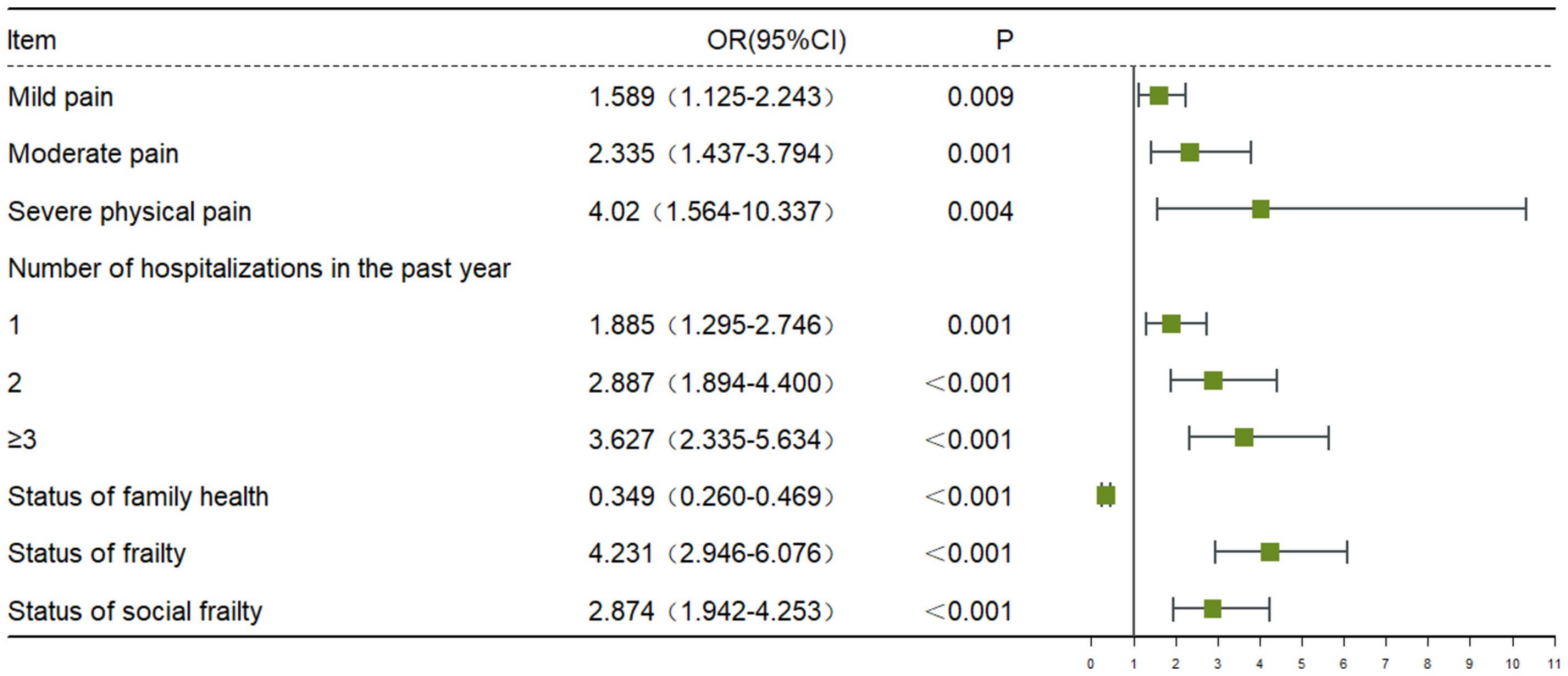
Binary logistic regression analysis of factors associated with depressive symptoms.

The influencing factors of depressive symptoms in older adults are shown in Table 2 and Figure 2. Depressive symptoms were more severe in older adults with mild body pain, moderate body pain, and severe body pain (OR = 1.589, 95% CI 1.125–2.243; OR = 2.335, 95% CI 1.437–3.794; OR = 4.020, 95% CI 1.564–10.337). The number of hospitalizations in the past year is a risk factor for depressive symptoms in older adults; older people who have been hospitalized once, twice and more than three times have a significantly increased risk of depressive symptoms, which was 1.885, 2.887 and 3.627 times higher than healthy people, respectively. The more severe the frailty in older adults, the higher the depressive symptom score (OR = 4.231, 95% CI 2.946–6.076). Social frailty in older adults increased the risk of depressive symptoms (OR = 2.874, 95% CI 1.942–4.253). Family health was a protective factor for depressive symptoms in older adults (OR = 2.867, 95% CI 2.134–3.851; Figure 3).

**Table 2:** Binary logistic regression analysis of factors associated with depressive symptoms.

Characteristics	B	S.E.	Wald	*p*	OR	(95%CI)
Sociodemographic						
Status of body pain			17.079	0.001		
Mild pain	0.463	0.176	6.911	0.009	1.589	1.125–2.243
Moderate pain	0.848	0.248	11.717	0.001	2.335	1.437–3.794
Severe physical pain	1.391	0.482	8.338	0.004	4.020	1.564–10.337
Number of hospitalizations in the past year			40.969	<0.001		
1	0.634	0.192	10.931	0.001	1.885	1.295–2.746
2	1.060	0.215	24.300	<0.001	2.887	1.894–4.400
≥3	1.289	0.225	32.887	<0.001	3.627	2.335–5.634
Status of family health	−1.053	0.151	48.942	<0.001	0.349	0.260–0.469
Status of frailty	1.442	0.185	60.992	<0.001	4.231	2.946–6.076
Status of social frailty	1.056	0.200	27.851	<0.001	2.874	1.942–4.253
Constant	−2.665	0.190	197.349	<0.001	0.070	

## Discussion

In recent years, with the acceleration of population aging in China, the prevalence of depressive symptoms in older adults has continued to increase, which has attracted widespread attention from more and more scholars. The purpose of this study was to explore the prevalence and influencing factors of depressive symptoms among older adults in China. In this cross-sectional study, we explored the prevalence of depressive symptoms and risk factors for depressive symptoms in 1175 older adults. The results showed that physical pain, hospitalizations in the past year, frailty, social frailty and poor family health were more likely to cause depressive symptoms in older adults than other factors. The research results provide a certain theoretical basis for the screening, prevention and psychological intervention of depressive symptoms in older adults.

In this cross-sectional study, we found that the prevalence of depressive symptoms with PHQ-9 total score ≥ 10 among Chinese older adults was 34.6%. The findings are consistent with some previous studies, including Zhou L et al. (43), who reported a prevalence of depressive symptoms of 35.19% among 4,771 older people in China. The prevalence of depressive symptoms among the older adults in this study was higher than the 20.36% prevalence rate of depressive symptoms among the community-dwelling older adults in Hainan, China (44), and the 16.2% prevalence rate of depressive symptoms among a Longitudinal Cohort Study (45), but lower than the reported 53% prevalence rate of depressive symptoms among the rural older adults in Anhui, China (46). The results of this study, like previous similar studies, suggesting that the depressive symptoms in older adults in China are serious, and the burden of depressive symptoms had been and would be gradually increase, placing a heavy burden on medical and health institutions. In addition, Yan Yu meng et al. used CHARLS data in 2015 and 2018 to study the prevalence of depressive symptoms among older adults in China. The results showed that the prevalence of depressive symptoms among older adults in China increased from 33.8% in 2015 to 50.6% in 2018 (47). Our results show that the prevalence of depressive symptoms among older adults in Anhui Province was different from that reported in other studies, which may be due to different assessment tools and survey times among the populations involved in studies conducted in different regions. It is worth mentioning that the PHQ-9 has been proven to be a reliable and valid screening tool for depressive symptoms, and it has been widely used in relevant cross-sectional studies on depressive symptoms among older adults in China. Therefore, the prevalence of depressive symptoms in older adults measured by the PHQ-9 still has a certain degree of reliability in this study.

Consistent with previous studies, our present study results demonstrated that frailty was significantly positively associated with the occurrence of depressive symptoms among older adults in China (48, 49). Previous a Mendelian randomization studyhave shown that a strong bidirectional relationship between depressive symptoms and frailty among older adults (48). Several potential reasons could explain the positive association between frailty and depressive symptoms in older adults. ① In epidemiological studies, frail older adults are often affected by higher levels of chronic, mild inflammatory markers, especially interleukin-6 (IL-6), which serves as a biomarker of depressive symptoms in older adults substances are thought to be more susceptible to geriatric depression (50). ② Frailty is a clinical syndrome in older adults characterized by weakness, slow gait, inactivity, exhaustion, and weight loss. As time goes on, older adults may have a sense of despair, worthlessness, and emptiness due to limited physiological functions, and may no longer be interested in things they were originally interested in, which may lead to symptoms of depression to a certain extent (51). We concluded that participants with frailty was at higher risk for depressive symptoms. Therefore, it is necessary to conduct regular frailty screening for older adults, and provide timely psychological comfort and psychological counseling to the frail older adults.

In our study, social frailty scores were positively correlated with depressive symptom scores, and these data suggest that social frailty might be a risk factor for depressive symptoms. The results of binary logistic regression analysis showed that participants social frailty were 2.874 times more likely to experience depressive symptoms than non-socially frailty participants. When older adults entered the second half of their lives, they tend to reduce their social participation activities to varying degrees due to age, chronic illness and other reasons. The dramatic change in social roles may result in less interaction with people, which may further cause some degree of mental health problems in older adults (52). According to the stress-buffering hypothesis, social activities may significantly reduce adverse psychological effects on individuals’ mental health through their buffering effect on stress levels (53). In addition, research have shown that group psychological support, friend-making activities, and social support interventions could have a positive impact on the mental health of older adults and help improve their social frailty of older adults (23). Therefore, it is recommended that mental health staff pay more attention to and dynamically assess the social frailty and depressive symptoms of older adults, enrich the social activities of older adults, and play an active role in social participation.

Interestingly, our findings showed that older adults with healthy families were 0.677 times less likely to experience depressive symptoms. In this study, family health was found to be a protective factor for depressive symptoms in older adults, indicating that family health plays an important role in improving mental health in older adults, this means that poor family health may lead to significant increases in depressive symptoms in older adults. Family is the essential environment for individual life. In traditional Chinese culture, family means “shelter” and is the primary basis for solving psychological problems (54). Therefore, family health is considered a health unit involving each family member and the entire family system (55). According to family systems theory, families provide a strong emotional foundation for maintaining supportive relationships among members, which helps promote psychological well-being (56). The family is both a resource and a priority group that plays a very critical preventive and therapeutic role throughout the life course of its members’ life course (57). Family health emphasizes health-related elements and closely links personal health with social health, which can enhance the family’s ability to obtain external resources. The study found that family health was significantly negatively correlated with frailty among Chinese older adults. Improving family health can increase individual health literacy and reverse frailty (29). Previous research has shown that the role of family is critical in predicting better health-related quality of life in older adults (58). The existence of family is an essential source of social support for older adults as they accept the aging process, and family health helps motivate older adults to participate in daily activities and improve self-esteem (59).

This study’s results revealed that physical pain is a risk factor for depressive symptoms in older adults, which is consistent with Lu L’s previous study (60). Possible explanations for this finding include: First, physical pain directly contributes to negative emotions and negative experiences in older adults. Secondly, chronic pain generally accompanies the occurrence of chronic diseases, which can lead to adverse consequences such as limited physical activity and high disability rates. The worry about adverse consequences can lead to an increase in depressive symptoms in older adults (61).

The results of this study indicate that the prevalence of depressive symptoms in older adults increases with the number of hospitalizations. One possible explanation for this research result may be related to chronic disease and its complications. Older adults who are hospitalized more often are more likely to have more and more severe chronic conditions, and these chronic conditions often carry serious associated complications with age. Increased hospitalizations may lead to increased frailty. In addition, older adult’s excessive worries about their physical health and the pressure of medical expenses would have a more profound negative impact on their mental health status, which may further induce depressive symptoms in older adults (62). Therefore, for older people with acute and chronic diseases, physical health status is one of the risk factors for depressive symptoms. The prevalence of depression in older adults might be further reduced by improving their health.

This study provides a specific scientific basis for the improvement of the theoretical framework related to depression in the older adults, the optimization of intervention strategies, and the formulation of public policies. This not only promotes cutting-edge research on older adult’s mental health but also provides specific guidance for achieving healthy aging.

## Strengths and limitations

Our study has strengths to this study. The PHQ-9 scale is well-known for being a simple and effective test for measuring depressive symptoms. However, some limitations of the current study cannot be ignored. First, cross-sectional designs cannot infer causal relationships among factors influencing depressive symptoms in older adults. Future large-sample multicenter prospective cohort studies are necessary to validate causal associations. Second, the PHQ-9 was designed to measure depressive symptoms and was not a diagnostic tool for depression. Third, self-reported scales may introduce information bias from participants and investigators, and subsequent cross-sectional studies need to introduce more objective measurement tools.

## Conclusion

Our data showed a high prevalence of depressive symptoms among Chinese older adults. Physical pain, number of hospitalizations in the past year, frailty, and social frailty were found to be significant predictors of depressive symptoms. At the same time, family health was a protective factor for depressive symptoms. These results indicate that it is critical to proactively and effectively identify mental health conditions in older adults. Our study contributes to the early identification and management of depressive symptoms in older adults, which may help prevent the possible harmful effects of depressive symptoms on the physical and mental health of older adults. Promote the health and well-being of the older adults and actively respond to the country’s strategic requirements for dealing with population aging.

## Data Availability

The raw data supporting the conclusions of this article will be made available by the authors, without undue reservation.
